# Improved dietary diversity of school adolescent girls in the context of urban Northwest Ethiopia: 2017

**DOI:** 10.1186/s13052-018-0490-0

**Published:** 2018-04-25

**Authors:** Samuel Mersha Birru, Amare Tariku, Aysheshim Kassahun Belew

**Affiliations:** 1 Gondar University Teaching and Referral Hospital, University of Gondar, Gondar, Ethiopia; 2 Department of Human Nutrition, Institute of Public Health, University of Gondar, Gondar, Ethiopia

**Keywords:** School adolescent girls, Dietary diversity, Ethiopia

## Abstract

**Background:**

Undiversified diet increases the vulnerability of adolescents for different nutritional problems. Therefore, this study assessed dietary and associated factors among school adolescent girls in Gondar city, northwest Ethiopia.

**Methods:**

A cross-sectional study was conducted from March to April 2017. Simple random sampling technique was used to select 778 adolescent school girls. A structured questionnaire was used to collect data. Multivariable logistic regression analysis was fitted to identify significant factors of dietary diversity. Adjusted Odds Ratio (AOR) with 95% confidence interval was used to show the strength of association, while a *P*-value< 0.05 of was used to declare the significance of association.

**Results:**

This finding indicated that adolescent girls who met minimum dietary diversity with were 75.4% (95%CI (72.3, 78.6). School type (AOR = 3.17, 95%CI: 1.90, 5.28) and residence (AOR = 2.0, 95%CI: 0.84, 3.01) were significantly associated with adequate dietary diversity.

**Conclusions:**

Dietary practice of adolescent girls is good in Gondar City Administration. School type and residence of adolescents were significantly associated with minimum dietary diversity. Thus, Special attention needs to be paid on government school and rural adolescents to improve the intake of variety diet.

## Background

Dietary diversity score (DDS) is defined as a number of individual food groups consumed over a given period of time [1]. It reflects quality diet at the household or individual level. In addition, DDS is measure of food security, nutrition information, early warning system and target of intervention at Global or national level [2, 3]. Mostly, monotonous staple diets lack essential micronutrients which lead to macro and micronutrient deficiencies, particularly in the most vulnerable group [4, 5].

Optimal nutrition is critical during adolescence as 50, 20 and 50% of adult weight, height, and skeletal mass are gained in this period [6–8]. However, 45–60% of adolescent girls are found with sub-optimal dietary intake [9] resulting in development of varied micronutrient deficiencies (Vitamin A, iron and iodine deficiencies) [5, 8, 10–12]. Similar finding showed in Ethiopian 29 and 30% of adolescent girls had thinness and anemia, respectively [13]. Furthermore, delayed puberty, contracted pelvis and unfavorable birth outcomes are noted in undernourished adolescent girls [14, 15].

Globally, Only 17% of adolescents had diversified diet [16]. Similarly, 23.5–50% [17, 18] of the Iranian, 11.2% of Zimbabwe [19] and 26.8% [4] Ethiopian Adolescents were reported as having adequate dietary diversity. Number of the researches documented that maternal education [4, 20], school type [20], occupation [20], nutritional knowledge [21], household food security [22], residence [4, 22] and wealth status [4, 20, 22] were associated with dietary diversity of adolescents. Considering the burden of sub-optimal dietary intake, promotion of adequate dietary diversity becomes one of the global concerns [23]. However, the level of adolescent’s dietary diversity is not well investigated in Ethiopia [4]. Therefore, this study assessed dietary diversity and associated factors among school adolescent girls in Gondar City Administration.

## Methods

A school-based quantitative cross-sectional study was conducted from March to April 2017 in Gondar City Administration; northwest Ethiopia. The City has 42 primary and 14 secondary and preparatory schools. All adolescent girls attending both private and governmental schools during the study period were considered as a source population.

A single population proportion formula was used to estimate sample size. Assumptions considered in sample size calculation were 26.8% as prevalence of adequate dietary diversity among adolescent high school girls in Gurage Zone [4], 95% confidence level, 1.5 design effect, 10% non-response rate and 4% degree of precision. Finally, a sample size of 778 was obtained. Schools were stratified into private and governmental schools and then ten schools were selected by lottery method. Simple random sampling technique was employed to choose samples using complete list of students as a sampling frame. Participants included in the study were proportional to the total students enrolled in private and governmental schools.

Concerning the data collection activity, interviewed based questionnaire was used to collect data. The tool was developed by reviewing different literatures, food and Agriculture Organization (FAO); FHI 360. Minimum Dietary Diversity for Women and health survey reports. Primarily the tool was prepared in English and translated to Amharic, the local language, and re-translated to English to check consistency of the questionnaire. Four data collectors (clinical nurses) and two BSc nurses as a supervisor were trained prior to data collection. The questionnaire was pretested among 39 school adolescent girls out of the study area. The training majorly encompasses dietary intake measurement and questionnaire administration techniques and ethical issues of the study.

The outcome, dietary diversity, was assessed using a standard tool suggested by Food and Agricultural Organization to measure women’s dietary diversity. Food consumed by adolescents was assessed through 24-h recall method and then food items were categorized into ten food groups. Dietary Diversity Score (DDS) was created as a summary measure of dietary intake, accordingly participants who had DDS of five and above were deemed as having adequate dietary diversity, whereas inadequate DDS was ascertained when they had less than five DDS [24].

EPI INFO version 3.5.3 and SPSS version 20 were used for data entry and analysis, respectively. A binary logistic regression model was fitted to show the effect of exposure variables on dietary diversity. A variable screening criteria of *P*-value less than 0.2 was used in the bivariate analysis to select candidate variables for the final model, multi-variable logistic regression analysis. In the adjusted analysis, independent variables with a *P*-value of < 0.05 were considered as independent factors associated with Dietary Diversity. Model fitness was checked using Hosmer-Lemeshow goodness of the fit test**.**

## Results

A total of 768 adolescent girls participated in the study with a response rate 98.7%. The mean age (±SD) of the adolescent was 15.49(± 1.93) years. Majority (79.3%) of respondents attended government schools. Substantial proportion (93.4%) of samples lived in urban settlements and 74.3% of received less than three meals per day. About 33.7% of respondents were stunted, whereas, only 2.5% were thin for their height (Table 1). Home gardening was reported by 16% of the study participants. Almost all (98.4 and 98.5%, respectively) households used water from improved sources and took less 30 min to fetch water in round trip (Table 2).

**Table 1:** Socio-demographic characteristics of school adolescent girls and their parents, Gondar City Administration, northwest Ethiopia, 2017 (*n* = 768)

Variables		Frequency	Percent
Age	Early	130	16.9
	Middle	402	52.3
	Late	236	30.7
Level of education	Primary	287	37.4
	High school	417	54.3
	Preparatory school	64	8.3
Place of residence	Urban	717	93.4
	Rural	51	6.6
Occupation of mother	Government employee	124	16.1
	Housewife	482	62.8
	Daily laborer	39	5.1
	Merchant	83	10.8
	Others	40	5.2
Media exposure	Exposed	608	79.2
	Not exposed	160	20.8
Food security	Secured	485	63.2
	In secured	283	36.8
Family wealth status	Rich	239	31.1
	Middle	287	37.4
	Poor	242	31.5
Maternal education	Unable to read and write	427	55.6
	Primary	117	15.2
	Secondary	123	16
	College and above	101	13.2
Father’s education	Unable to read and write	370	48.2
	Primary	96	12.5
	Secondary	125	16.3
	College and above	177	23

**Table 2:** Environmental characteristics of school adolescent girls in Gondar City Administration, Northwest Ethiopia, 2017

Variables		Frequency	Percent
Availability of home gardening	Yes	123	16.0
	No	645	84.0
Source of drinking water	Improved	756	98.4
	Unimproved	12	1.6
Wate water treatment	yes	572	74.5
	No	196	25.5
Availability of home latrine	yes	607	79.0
	No	161	21.0
Hand washing after toilet	Yes	757	98.6
	No	11	1.4

**Fig. 1:**
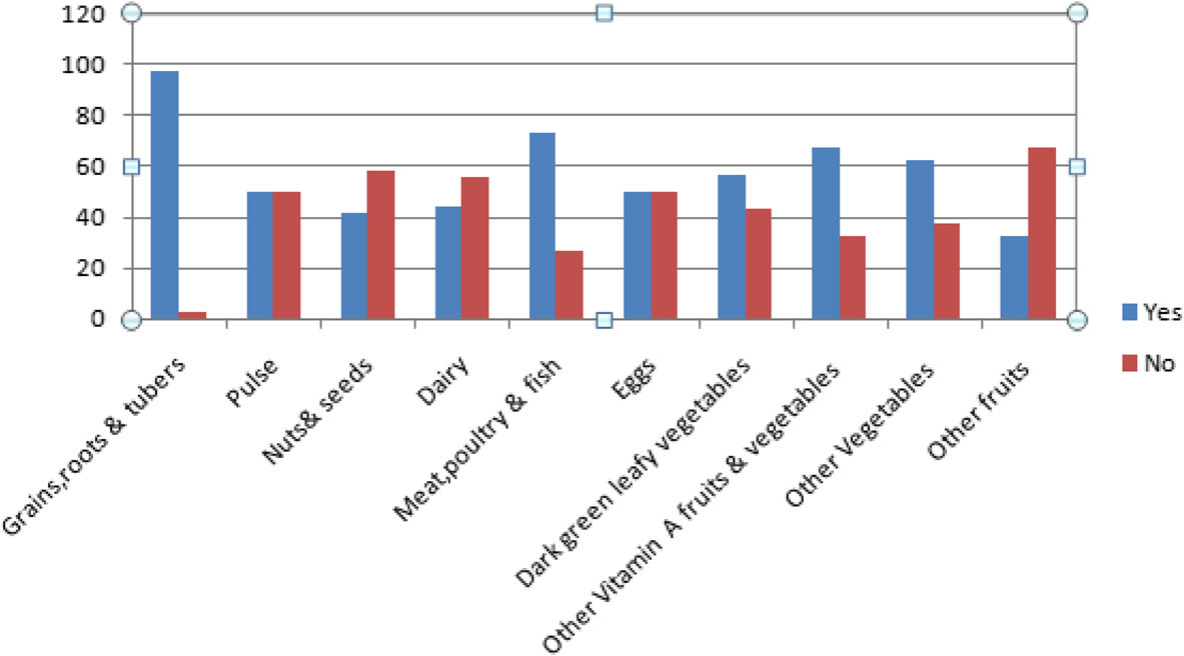
Types of food groups consumed among adolescent girls in Gondar City, Northwest, Ethiopia, 2017

Overall, 75.4%(95% CI: 72.3, 78.6) adolescent girls had adequate dietary diversity. Moreover, the mean dietary diversity score of participants was 5.76 ± 1.81. Majority (97.7%) of adolescent girls consumed starchy staples (grains, roots and tuber). However, only 32.4% ate fruits (Fig. 1).

The logistic regression output illustrated that school type and maternal occupation were significantly associated with dietary diversity. Adolescents who attended private schools 3.2 (AOR =3:17; 95% CI: 1.1.9, 5.28) and lived in urban areas (AOR = 2.0; 95% CI: 0.84, 3.01) had increased odds diversified diet (Table 3).

**Table 3:** Bivariate and multivariable logistic regression output showing that factors associated with dietary diversityamong school adolescent girls, Gondar City Administration, northwest Ethiopia, 2017

Variables	Dietary diversity		Crude Odds Ratio with 95% C)	Adjusted Odds Ratio with 95% CI
	Adequate	Not adequate		
Age of adolescent				
Early	90 (69.2%)	40 (30.8%)	1	1
Middle	216 (53.7%)	186 (46.3%)	0.52 (0.34,0.79)	1.25 (0.71,2.20)
Late	93 (39.4%)	143 (60.6%)	0.29 (0.18,0.46)	1.01 (0.51,2.01)
School type				
Government	280 (45.8%)	332 (54.2%)	1	1
Private	119 (76.3%)	37 (23.7%)	3.81 (2.55,5.70)	3.17 (1.90,5.28)^a^
Educational status of father				
Informal education	173 (46.8%)	197 (53.2%)	1	1
Primary	44 (49.9%)	52 (46.1%)	0.96 (0.61,1.51)	0.81 (0.50,1.31)
Secondary	77 (61.6%)	48 (38.4%)	1.83 (1.20,2.76)	1.27 (0.77,2.09)
College and above	105 (59.3%)	72 (40.7%)	1.66 (1.12,2.39)	0.98 (0.60,1.64)
Educational status of mothers				
Informal education	193 (45.2%)	234 (54.8%)	1	1
Primary	69 (59%)	48 (41%)	1.74 (1.15,2.64)	1.24 (0.78,1.96)
Secondary	72 (58.5%)	51 (41.5%)	1.71 (1.14,2.57)	1.05 (0.62,1.79)
College and above	65 (64.4%)	36 (35.6%)	2.19 (1.40,3.43)	1.38 (0.69,2.76)
Occupation of the mothers				
Government employee	69 (55.6%)	55 (44.4%)	1.39 (0.68,2.83)	1.61 (0.74,3.53)
Housewife	241 (50%)	241 (50%)	1.10 (0.58,2.11)	1.65 (0.81,3.33)
Daily laborer	17 (43.6%)	22 (56.4%)	0.85 (0.35,2.07)	1.26 (0.49,3.29)
Merchant	53 (63.9%)	30 (36.1%)	1.95 (0.91,4.20)	2.42 (1.06,5.53)
Others	19 (47.5%)	21 (52.5%)	1	1
Wealth status				
1st quintile	75 (45.5%)	90 (54.5%)	0.52(0.34,0.79)	0.78 (0.47,1.29)
2nd quintile	100 (47.2%)	112 (52.8%)	0.55 (0.37,0.82)	0.80 (0.51,1.25)
3rd quintile	106 (53%)	94 (47%)	0.70 (0.47,1.04)	0.85 (0.54, 1.31)
4th quintile	118 (61.8%)	73 (38.2%)	1	1
Availability of latrine				
Yes	324 (53.4%)	283 (46.6%)	1.31 (0.93,1.86)	0.88 (0.60,1.32)
No	75 (46.6%)	86 (53.4%)	1	1
Stunting				
Stunted	115 (44.4%)	144 (55.6%)	1	1
Normal	284 (55.8%)	225 (44.2%)	1.58 (1.17, 2.14)	1.00 (0.70, 1.42
Residence				
Urban	380 (53%)	337 (47%)	1.90 (1.06, 3.41)	2.00 (0.84, 3.01)^a^
Rural	19 (37.3%)	32 (62.7%)	1	1
Respondents educational status				
5–8	184 (64.1%)	103 (35.9%)	1	1
9–10	197 (47.2%)	220 (52.8%)	0.50 (0.37, 0.68)	0.69 (0.46, 1.03)
11–12	18 (28.5%)	46 (71.9%)	0.22 (0.12, 0.40)	0.23 (0.11, 0.50)

^a^indicate significant at *p* value less than 0.05 in multivariable logistic analysis

## Discussion

This study illustrated that three-fourth (75.4%) of adolescent girls had diversified diet. The finding was higher than the reports of developing countries, including Iran (26.55%) [25], Zimbabwe (11.2%) [26], Adama City (41.2%) [27], Gurage Zone (26.8%) [4], Amhara region (21.8%) [28]. The high prevalence of adequate dietary diversity in this study area could be due to the nature of the study. Almost all (93.4%) of adolescent girls were included from the urban kebeles and enrolled in schools. Such source of discrepancies could explain increased proportion of diversified diet. In addition, do not have information about better dietary intake habits [20]. However, the result was slightly lower than the study conducted in Agarfa, Ethiopia (80.4%) [29]. This study used five food groups as cut-off point to determine adequate diversity, whereas the previous study considered four food groups. Use of low cut-off might inflate the prevalence of diversified diet in the former study. Obviously, dietary habit of developing nations is entirely depends on starchy staples [30]. This study also confirmed that almost all (97.7%) of participants consumed grains, root and tubers. This result was supported with the previous local report [4].

The result of multivariate logistic regression analysis showed that adolescent girls from private schools had higher odds of diversified diet compared to those who attended governmental schools. In Ethiopia context, students enrolled in private schools are majorly from better-off families hence, poor families usually do not afford monthly school fee. High household socio-economic status is key to enhance household and individual dietary diversity [25, 31, 32]. Adolescents living in better-off households could have improved nutrition information access because of better availability of media source [33].

Lastly, the odds of diversified diet were high in adolescent who lived in urban areas compared to those who lived in rural areas. This finding was supported by researches elsewhere [4, 23, 34, 35] conduct in, China children [36], Mali [34], Gurage [4] and Jimma Zone [22]. In fact, food security, socio-economic status and access to variety of food and information are higher in the urban settlements than the rural areas [22, 35, 37, 38]. This might explain the observed difference in dietary diversity with place of residence. The study attempted to show dietary diversity in the most vulnerable group of the population representing the rural northwest Ethiopia, but, some of the limitations of this study should be taken into consideration. First, the study did not consider the quantity of food consumed by the adolescent and single 24 h recall did not indicate the usual dietary habit of the adolescent. There might be social desirability bias in responding type food given to children and recall bias.

## Conclusions

In summary, the prevalence of adequate dietary diversity is high in Gondar City Administration. Type of school and place of residence were significantly associated with adolescent’s dietary diversity. Hence, efforts focusing to improve dietary diversity should give special attention to government schools and the rural adolescents.

## Abbreviations

AOR: Adjusted Odds Ratio
BSC: Bachelors of Science
CI: Confidence Interval
COR: Crud Odds Ratio
DDS: Dietary Diversity Score
FAO: Food and Agriculture Organization
SPSS: Statistical Package for Social Sciences
